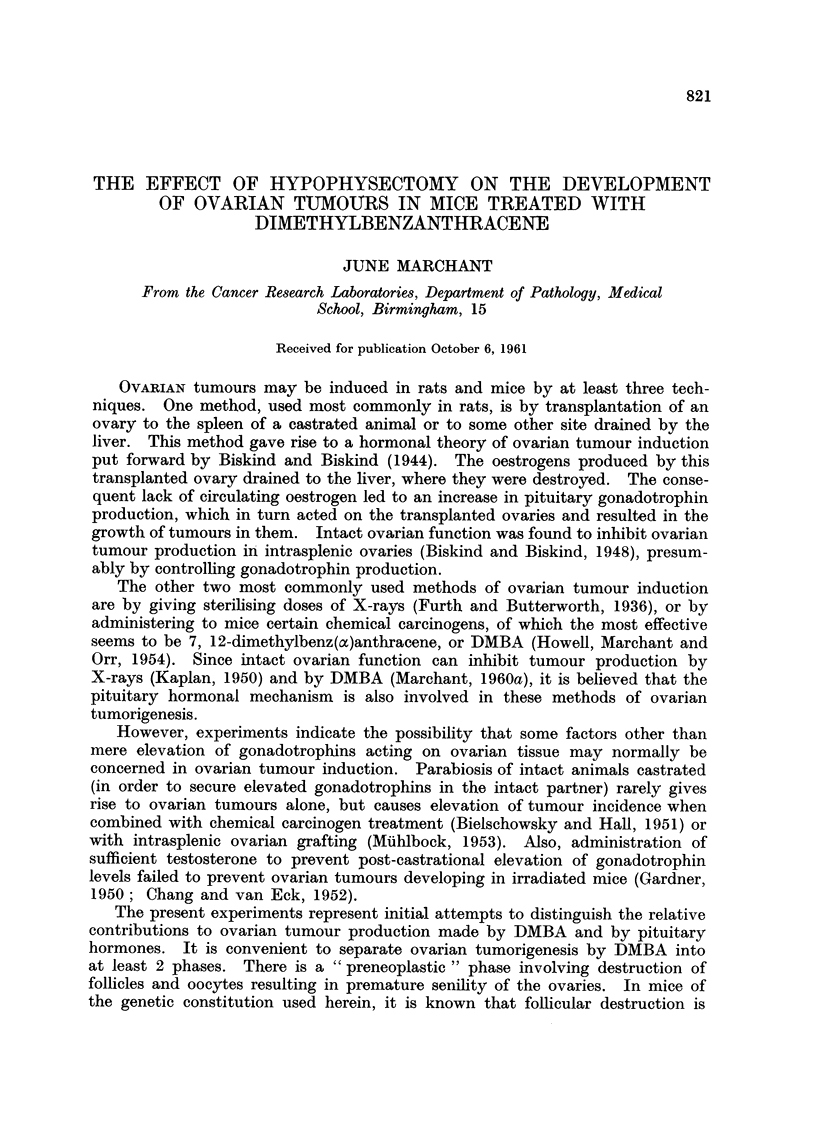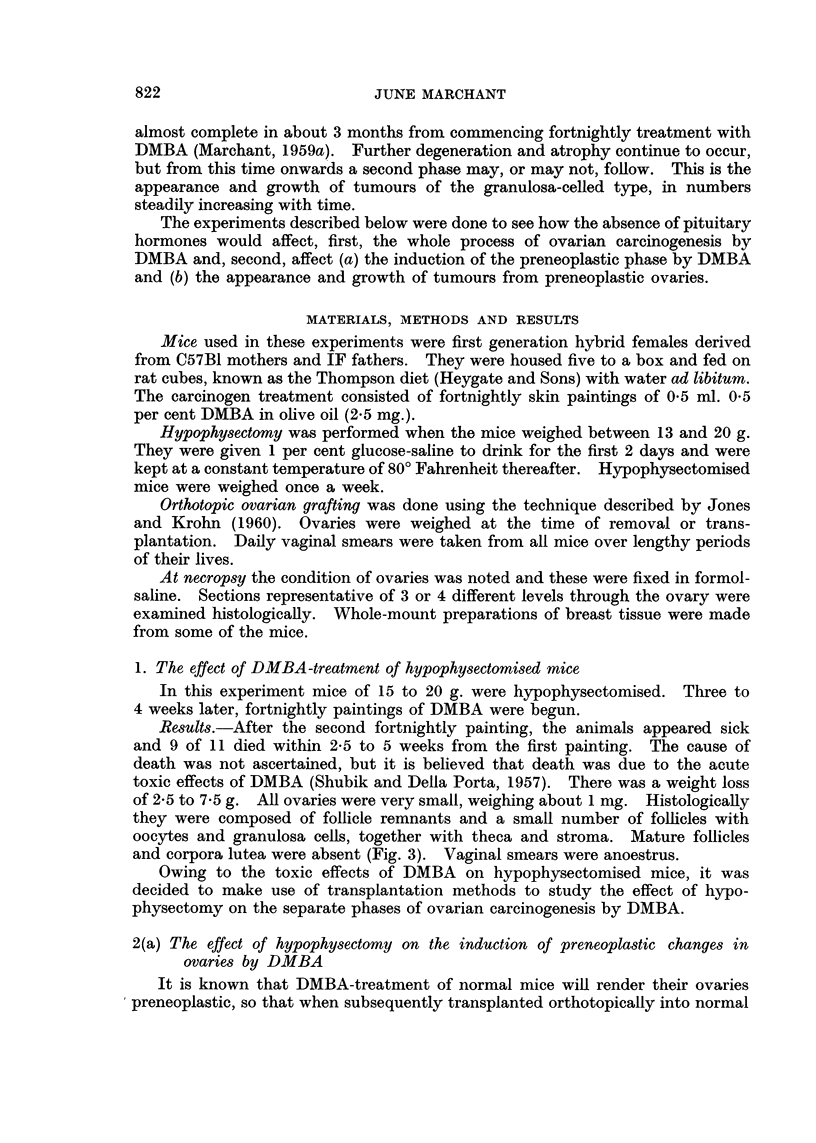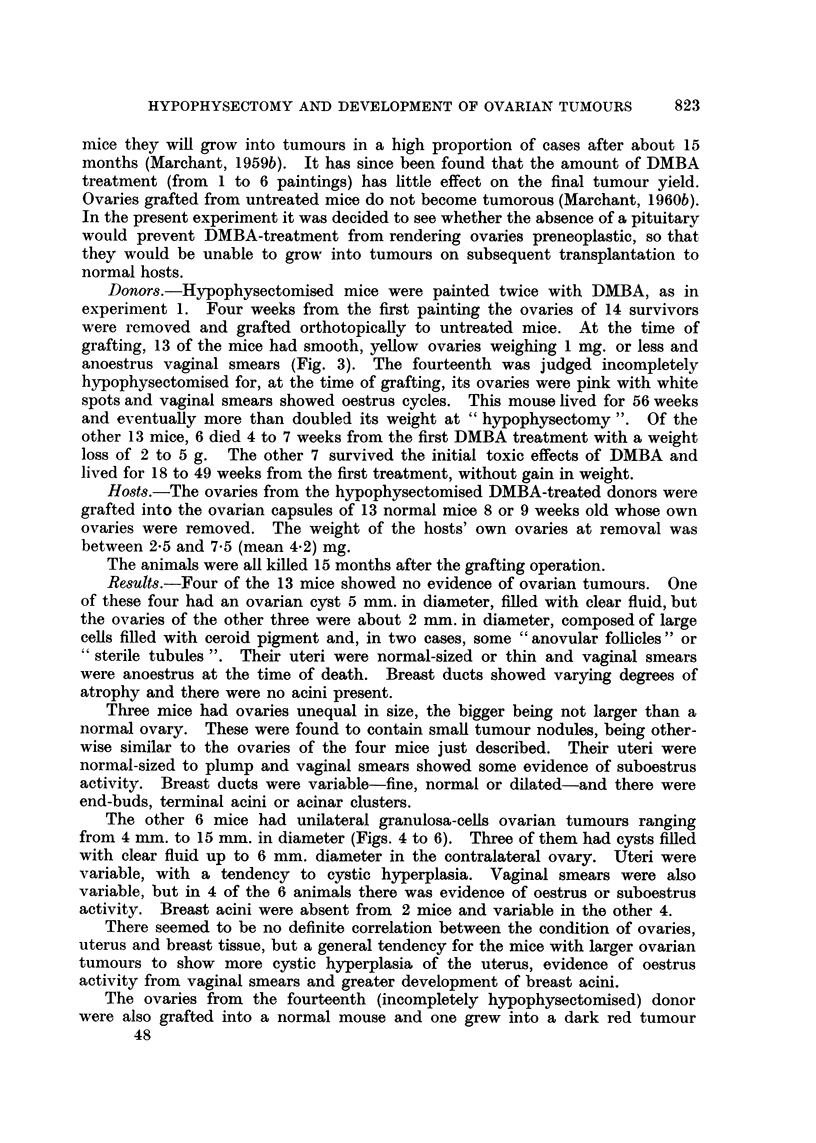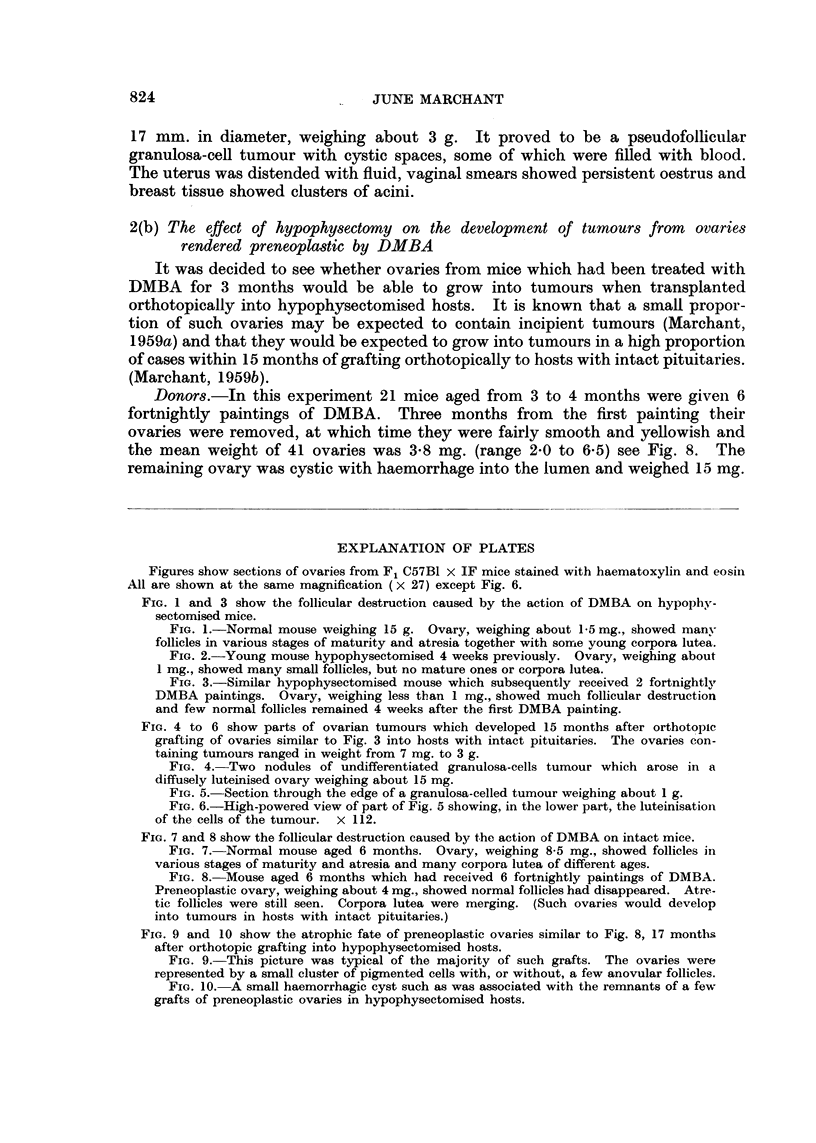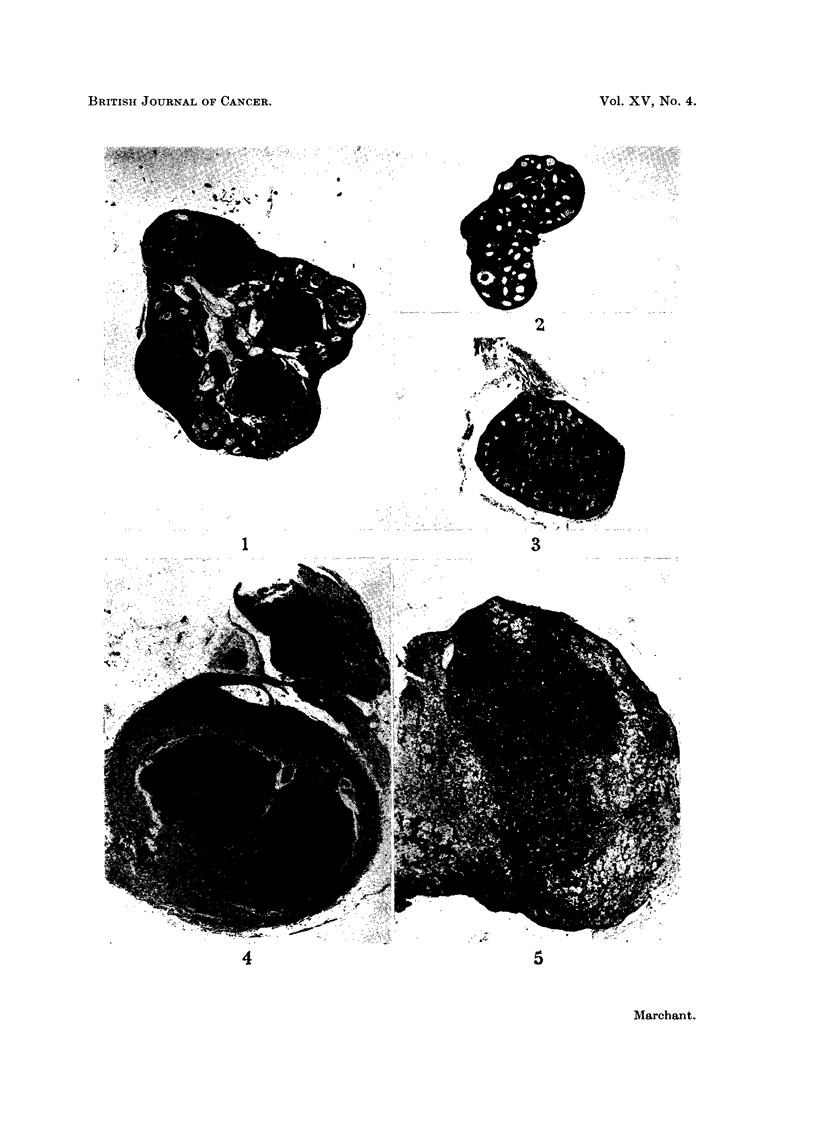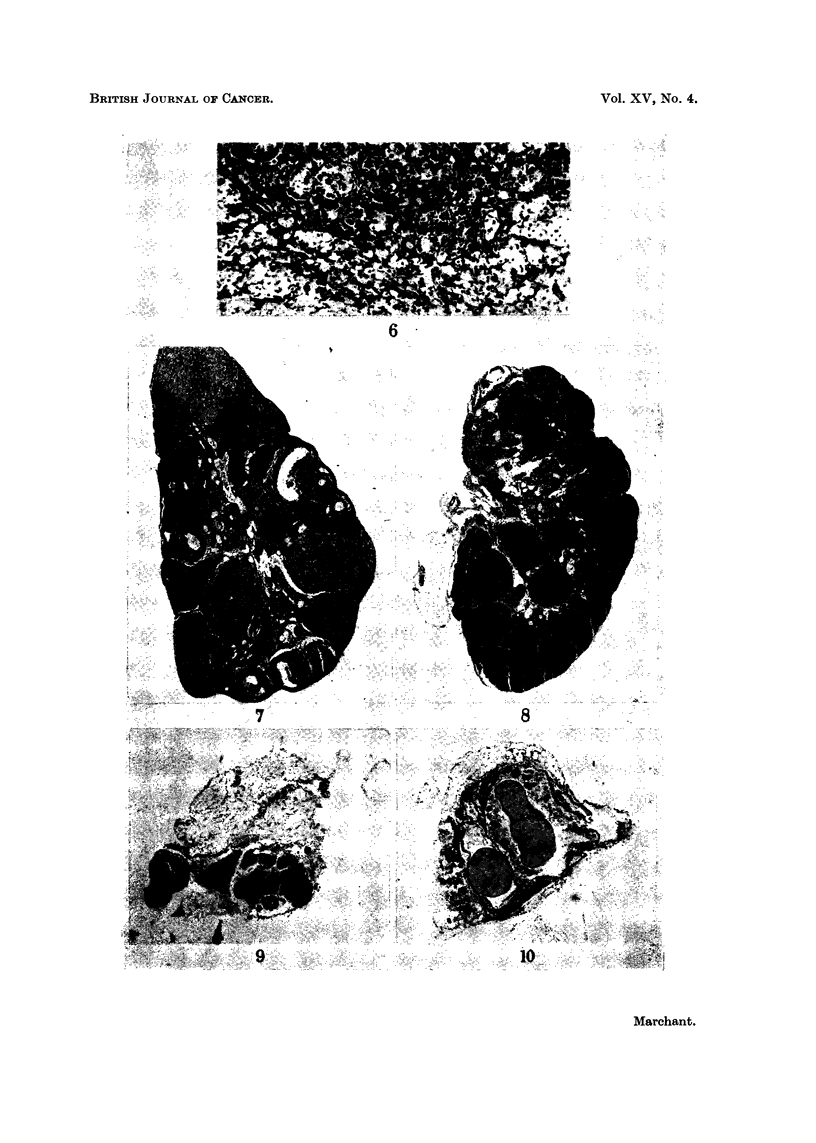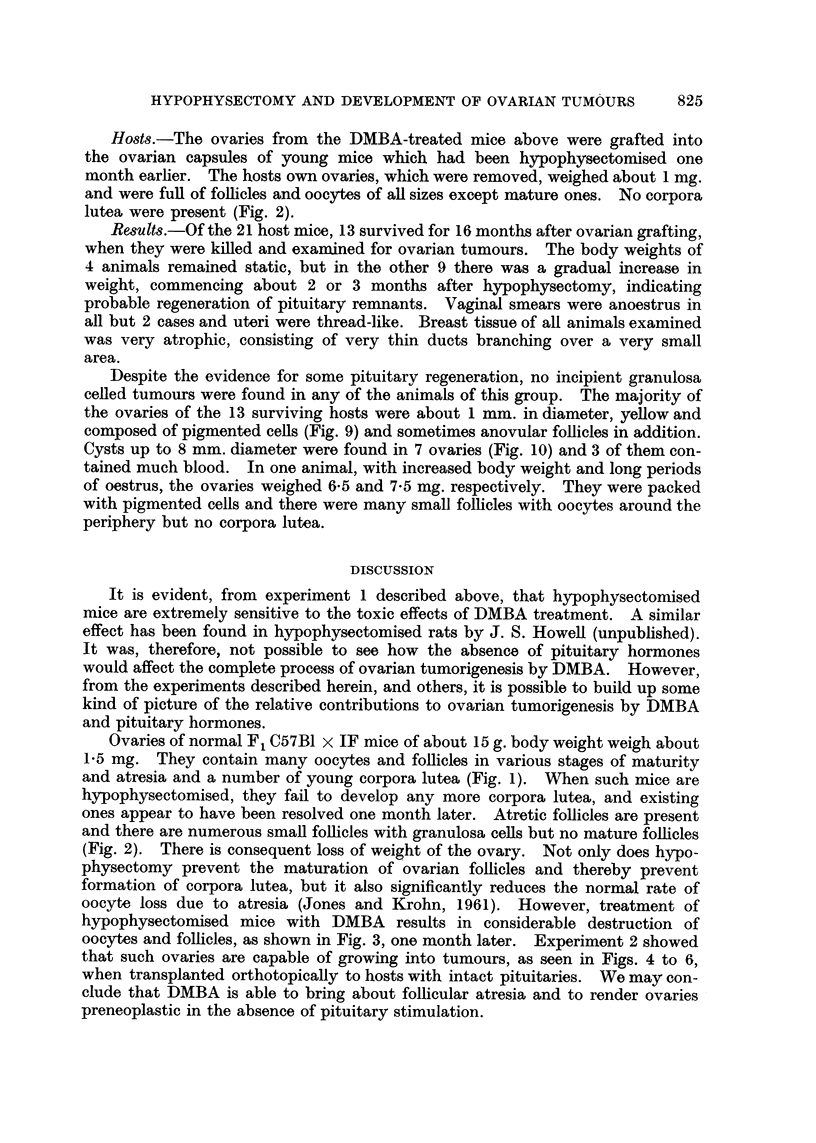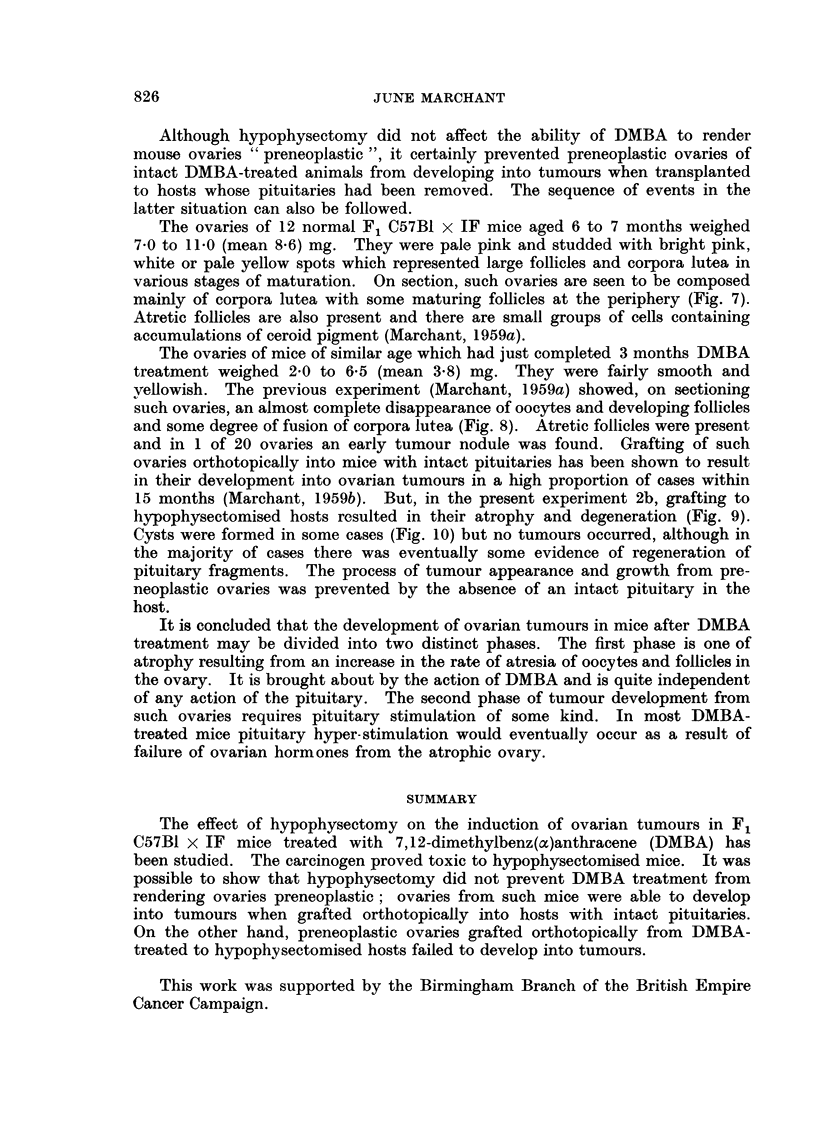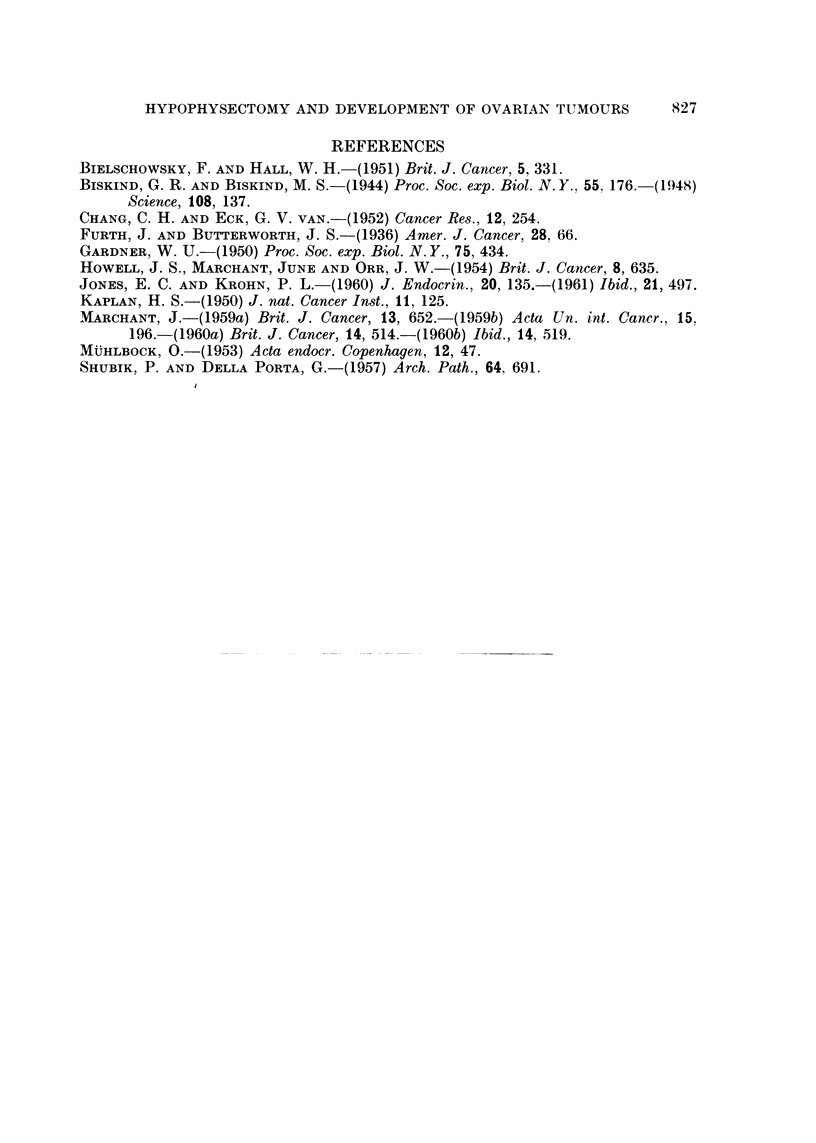# The Effect of Hypophysectomy on the Development of Ovarian Tumours in Mice Treated with Dimethylbenzanthracene

**DOI:** 10.1038/bjc.1961.94

**Published:** 1961-12

**Authors:** June Marchant

## Abstract

**Images:**


### THE EFFECT OF HYPOPHYSECTOMY ON THE DEVELOPMENT OF OVARIAN TUMOURS IN MICE TREATED WITH DIMETHYLBENZANTHRACENE

JUNE MARCHANT

*From the Cancer Research Laboratories, Department of Pathology, Medical School, Birmingham,* 15

Received for publication October 6, 1961

OVARIAN tumours may be induced in rats and mice by at least three techniques. One method, used most commonly in rats, is by transplantation of an ovary to the spleen of a castrated animal or to some other site drained by the liver. This method gave rise to a hormonal theory of ovarian tumour induction put forward by Biskind and Biskind (1944). The oestrogens produced by this transplanted ovary drained to the liver, where they were destroyed. The consequent lack of circulating oestrogen led to an increase in pituitary gonadotrophin production, which in turn acted on the transplanted ovaries and resulted in the growth of tumours in them. Intact ovarian function was found to inhibit ovarian tumour production in intrasplenic ovaries (Biskind and Biskind, 1948), presumably by controlling gonadotrophin production.

The other two most commonly used methods of ovarian tumour induction are by giving sterilising doses of X-rays (Furth and Butterworth, 1936), or by administering to mice certain chemical carcinogens, of which the most effective seems to be 7, 12-dimethylbenz($\alpha$)anthracene, or DMBA (Howell, Marchant and Orr, 1954). Since intact ovarian function can inhibit tumour production by X-rays (Kaplan, 1950) and by DMBA (Marchant, 1960*a*), it is believed that the pituitary hormonal mechanism is also involved in these methods of ovarian tumorigenesis.

However, experiments indicate the possibility that some factors other than mere elevation of gonadotrophins acting on ovarian tissue may normally be concerned in ovarian tumour induction. Parabiosis of intact animals castrated (in order to secure elevated gonadotrophins in the intact partner) rarely gives rise to ovarian tumours alone, but causes elevation of tumour incidence when combined with chemical carcinogen treatment (Bielschowsky and Hall, 1951) or with intrasplenic ovarian grafting (Mühlbock, 1953). Also, administration of sufficient testosterone to prevent post-castrational elevation of gonadotrophin levels failed to prevent ovarian tumours developing in irradiated mice (Gardner, 1950 ; Chang and van Eck, 1952).

The present experiments represent initial attempts to distinguish the relative contributions to ovarian tumour production made by DMBA and by pituitary hormones. It is convenient to separate ovarian tumorigenesis by DMBA into at least 2 phases. There is a " preneoplastic " phase involving destruction of follicles and oocytes resulting in premature senility of the ovaries. In mice of the genetic constitution used herein, it is known that follicular destruction is almost complete in about 3 months from commencing fortnightly treatment with DMBA (Marchant, 1959*a*). Further degeneration and atrophy continue to occur, but from this time onwards a second phase may, or may not, follow. This is the appearance and growth of tumours of the granulosa-celled type, in numbers steadily increasing with time.

The experiments described below were done to see how the absence of pituitary hormones would affect, first, the whole process of ovarian carcinogenesis by DMBA and, second, affect (*a*) the induction of the preneoplastic phase by DMBA and (*b*) the appearance and growth of tumours from preneoplastic ovaries.

### MATERIALS, METHODS AND RESULTS

*Mice* used in these experiments were first generation hybrid females derived from C57Bl mothers and IF fathers. They were housed five to a box and fed on rat cubes, known as the Thompson diet (Heygate and Sons) with water *ad libitum*. The carcinogen treatment consisted of fortnightly skin paintings of 0·5 ml. 0·5 per cent DMBA in olive oil (2·5 mg.).

*Hypophysectomy* was performed when the mice weighed between 13 and 20 g. They were given 1 per cent glucose-saline to drink for the first 2 days and were kept at a constant temperature of 80° Fahrenheit thereafter. Hypophysectomised mice were weighed once a week.

*Orthotopic ovarian grafting* was done using the technique described by Jones and Krohn (1960). Ovaries were weighed at the time of removal or transplantation. Daily vaginal smears were taken from all mice over lengthy periods of their lives.

*At necropsy* the condition of ovaries was noted and these were fixed in formol-saline. Sections representative of 3 or 4 different levels through the ovary were examined histologically. Whole-mount preparations of breast tissue were made from some of the mice.

### 1. *The effect of DMBA-treatment of hypophysectomised mice*

In this experiment mice of 15 to 20 g. were hypophysectomised. Three to 4 weeks later, fortnightly paintings of DMBA were begun.

*Results*.—After the second fortnightly painting, the animals appeared sick and 9 of 11 died within 2·5 to 5 weeks from the first painting. The cause of death was not ascertained, but it is believed that death was due to the acute toxic effects of DMBA (Shubik and Della Porta, 1957). There was a weight loss of 2·5 to 7·5 g. All ovaries were very small, weighing about 1 mg. Histologically they were composed of follicle remnants and a small number of follicles with oocytes and granulosa cells, together with theca and stroma. Mature follicles and corpora lutea were absent (Fig. 3). Vaginal smears were anoestrus.

Owing to the toxic effects of DMBA on hypophysectomised mice, it was decided to make use of transplantation methods to study the effect of hypophysectomy on the separate phases of ovarian carcinogenesis by DMBA.

### 2(a) *The effect of hypophysectomy on the induction of preneoplastic changes in ovaries by DMBA*

It is known that DMBA-treatment of normal mice will render their ovaries preneoplastic, so that when subsequently transplanted orthotopically into normal mice they will grow into tumours in a high proportion of cases after about 15 months (Marchant, 1959*b*). It has since been found that the amount of DMBA treatment (from 1 to 6 paintings) has little effect on the final tumour yield. Ovaries grafted from untreated mice do not become tumorous (Marchant, 1960*b*). In the present experiment it was decided to see whether the absence of a pituitary would prevent DMBA-treatment from rendering ovaries preneoplastic, so that they would be unable to grow into tumours on subsequent transplantation to normal hosts.

*Donors.*—Hypophysectomised mice were painted twice with DMBA, as in experiment 1. Four weeks from the first painting the ovaries of 14 survivors were removed and grafted orthotopically to untreated mice. At the time of grafting, 13 of the mice had smooth, yellow ovaries weighing 1 mg. or less and anoestrus vaginal smears (Fig. 3). The fourteenth was judged incompletely hypophysectomised for, at the time of grafting, its ovaries were pink with white spots and vaginal smears showed oestrus cycles. This mouse lived for 56 weeks and eventually more than doubled its weight at " hypophysectomy ". Of the other 13 mice, 6 died 4 to 7 weeks from the first DMBA treatment with a weight loss of 2 to 5 g. The other 7 survived the initial toxic effects of DMBA and lived for 18 to 49 weeks from the first treatment, without gain in weight.

*Hosts.*—The ovaries from the hypophysectomised DMBA-treated donors were grafted into the ovarian capsules of 13 normal mice 8 or 9 weeks old whose own ovaries were removed. The weight of the hosts' own ovaries at removal was between 2·5 and 7·5 (mean 4·2) mg.

The animals were all killed 15 months after the grafting operation.

*Results.*—Four of the 13 mice showed no evidence of ovarian tumours. One of these four had an ovarian cyst 5 mm. in diameter, filled with clear fluid, but the ovaries of the other three were about 2 mm. in diameter, composed of large cells filled with ceroid pigment and, in two cases, some "anovular follicles" or " sterile tubules ". Their uteri were normal-sized or thin and vaginal smears were anoestrus at the time of death. Breast ducts showed varying degrees of atrophy and there were no acini present.

Three mice had ovaries unequal in size, the bigger being not larger than a normal ovary. These were found to contain small tumour nodules, being otherwise similar to the ovaries of the four mice just described. Their uteri were normal-sized to plump and vaginal smears showed some evidence of suboestrus activity. Breast ducts were variable—fine, normal or dilated—and there were end-buds, terminal acini or acinar clusters.

The other 6 mice had unilateral granulosa-cells ovarian tumours ranging from 4 mm. to 15 mm. in diameter (Figs. 4 to 6). Three of them had cysts filled with clear fluid up to 6 mm. diameter in the contralateral ovary. Uteri were variable, with a tendency to cystic hyperplasia. Vaginal smears were also variable, but in 4 of the 6 animals there was evidence of oestrus or suboestrus activity. Breast acini were absent from 2 mice and variable in the other 4.

There seemed to be no definite correlation between the condition of ovaries, uterus and breast tissue, but a general tendency for the mice with larger ovarian tumours to show more cystic hyperplasia of the uterus, evidence of oestrus activity from vaginal smears and greater development of breast acini.

The ovaries from the fourteenth (incompletely hypophysectomised) donor were also grafted into a normal mouse and one grew into a dark red tumour

17 mm. in diameter, weighing about 3 g. It proved to be a pseudofollicular granulosa-cell tumour with cystic spaces, some of which were filled with blood. The uterus was distended with fluid, vaginal smears showed persistent oestrus and breast tissue showed clusters of acini.

2(b) *The effect of hypophysectomy on the development of tumours from ovaries rendered preneoplastic by DMBA*

It was decided to see whether ovaries from mice which had been treated with DMBA for 3 months would be able to grow into tumours when transplanted orthotopically into hypophysectomised hosts. It is known that a small proportion of such ovaries may be expected to contain incipient tumours (Marchant, 1959*a*) and that they would be expected to grow into tumours in a high proportion of cases within 15 months of grafting orthotopically to hosts with intact pituitaries. (Marchant, 1959*b*).

*Donors.*—In this experiment 21 mice aged from 3 to 4 months were given 6 fortnightly paintings of DMBA. Three months from the first painting their ovaries were removed, at which time they were fairly smooth and yellowish and the mean weight of 41 ovaries was 3·8 mg. (range 2·0 to 6·5) see Fig. 8. The remaining ovary was cystic with haemorrhage into the lumen and weighed 15 mg.

---

EXPLANATION OF PLATES

Figures show sections of ovaries from $F_1$ C57Bl × IF mice stained with haematoxylin and eosin All are shown at the same magnification (× 27) except Fig. 6.

FIG. 1 and 3 show the follicular destruction caused by the action of DMBA on hypophysectomised mice.

FIG. 1.—Normal mouse weighing 15 g. Ovary, weighing about 1·5 mg., showed many follicles in various stages of maturity and atresia together with some young corpora lutea.

FIG. 2.—Young mouse hypophysectomised 4 weeks previously. Ovary, weighing about 1 mg., showed many small follicles, but no mature ones or corpora lutea.

FIG. 3.—Similar hypophysectomised mouse which subsequently received 2 fortnightly DMBA paintings. Ovary, weighing less than 1 mg., showed much follicular destruction and few normal follicles remained 4 weeks after the first DMBA painting.

FIG. 4 to 6 show parts of ovarian tumours which developed 15 months after orthotopic grafting of ovaries similar to Fig. 3 into hosts with intact pituitaries. The ovaries containing tumours ranged in weight from 7 mg. to 3 g.

FIG. 4.—Two nodules of undifferentiated granulosa-cells tumour which arose in a diffusely luteinised ovary weighing about 15 mg.

FIG. 5.—Section through the edge of a granulosa-celled tumour weighing about 1 g.

FIG. 6.—High-powered view of part of Fig. 5 showing, in the lower part, the luteinisation of the cells of the tumour. × 112.

FIG. 7 and 8 show the follicular destruction caused by the action of DMBA on intact mice.

FIG. 7.—Normal mouse aged 6 months. Ovary, weighing 8·5 mg., showed follicles in various stages of maturity and atresia and many corpora lutea of different ages.

FIG. 8.—Mouse aged 6 months which had received 6 fortnightly paintings of DMBA. Preneoplastic ovary, weighing about 4 mg., showed normal follicles had disappeared. Atretic follicles were still seen. Corpora lutea were merging. (Such ovaries would develop into tumours in hosts with intact pituitaries.)

FIG. 9 and 10 show the atrophic fate of preneoplastic ovaries similar to Fig. 8, 17 months after orthotopic grafting into hypophysectomised hosts.

FIG. 9.—This picture was typical of the majority of such grafts. The ovaries were represented by a small cluster of pigmented cells with, or without, a few anovular follicles.

FIG. 10.—A small haemorrhagic cyst such as was associated with the remnants of a few grafts of preneoplastic ovaries in hypophysectomised hosts.

1

2

3

4

5

Marchant.

6

7

8

9

10

*Hosts.*—The ovaries from the DMBA-treated mice above were grafted into the ovarian capsules of young mice which had been hypophysectomised one month earlier. The hosts own ovaries, which were removed, weighed about 1 mg. and were full of follicles and oocytes of all sizes except mature ones. No corpora lutea were present (Fig. 2).

*Results.*—Of the 21 host mice, 13 survived for 16 months after ovarian grafting, when they were killed and examined for ovarian tumours. The body weights of 4 animals remained static, but in the other 9 there was a gradual increase in weight, commencing about 2 or 3 months after hypophysectomy, indicating probable regeneration of pituitary remnants. Vaginal smears were anoestrus in all but 2 cases and uteri were thread-like. Breast tissue of all animals examined was very atrophic, consisting of very thin ducts branching over a very small area.

Despite the evidence for some pituitary regeneration, no incipient granulosa celled tumours were found in any of the animals of this group. The majority of the ovaries of the 13 surviving hosts were about 1 mm. in diameter, yellow and composed of pigmented cells (Fig. 9) and sometimes anovular follicles in addition. Cysts up to 8 mm. diameter were found in 7 ovaries (Fig. 10) and 3 of them contained much blood. In one animal, with increased body weight and long periods of oestrus, the ovaries weighed 6·5 and 7·5 mg. respectively. They were packed with pigmented cells and there were many small follicles with oocytes around the periphery but no corpora lutea.

### DISCUSSION

It is evident, from experiment 1 described above, that hypophysectomised mice are extremely sensitive to the toxic effects of DMBA treatment. A similar effect has been found in hypophysectomised rats by J. S. Howell (unpublished). It was, therefore, not possible to see how the absence of pituitary hormones would affect the complete process of ovarian tumorigenesis by DMBA. However, from the experiments described herein, and others, it is possible to build up some kind of picture of the relative contributions to ovarian tumorigenesis by DMBA and pituitary hormones.

Ovaries of normal $F_1$ C57Bl × IF mice of about 15 g. body weight weigh about 1·5 mg. They contain many oocytes and follicles in various stages of maturity and atresia and a number of young corpora lutea (Fig. 1). When such mice are hypophysectomised, they fail to develop any more corpora lutea, and existing ones appear to have been resolved one month later. Atretic follicles are present and there are numerous small follicles with granulosa cells but no mature follicles (Fig. 2). There is consequent loss of weight of the ovary. Not only does hypophysectomy prevent the maturation of ovarian follicles and thereby prevent formation of corpora lutea, but it also significantly reduces the normal rate of oocyte loss due to atresia (Jones and Krohn, 1961). However, treatment of hypophysectomised mice with DMBA results in considerable destruction of oocytes and follicles, as shown in Fig. 3, one month later. Experiment 2 showed that such ovaries are capable of growing into tumours, as seen in Figs. 4 to 6, when transplanted orthotopically to hosts with intact pituitaries. We may conclude that DMBA is able to bring about follicular atresia and to render ovaries preneoplastic in the absence of pituitary stimulation.

Although hypophysectomy did not affect the ability of DMBA to render mouse ovaries " preneoplastic ", it certainly prevented preneoplastic ovaries of intact DMBA-treated animals from developing into tumours when transplanted to hosts whose pituitaries had been removed. The sequence of events in the latter situation can also be followed.

The ovaries of 12 normal $F_1$ C57Bl × IF mice aged 6 to 7 months weighed 7·0 to 11·0 (mean 8·6) mg. They were pale pink and studded with bright pink, white or pale yellow spots which represented large follicles and corpora lutea in various stages of maturation. On section, such ovaries are seen to be composed mainly of corpora lutea with some maturing follicles at the periphery (Fig. 7). Atretic follicles are also present and there are small groups of cells containing accumulations of ceroid pigment (Marchant, 1959*a*).

The ovaries of mice of similar age which had just completed 3 months DMBA treatment weighed 2·0 to 6·5 (mean 3·8) mg. They were fairly smooth and yellowish. The previous experiment (Marchant, 1959*a*) showed, on sectioning such ovaries, an almost complete disappearance of oocytes and developing follicles and some degree of fusion of corpora lutea (Fig. 8). Atretic follicles were present and in 1 of 20 ovaries an early tumour nodule was found. Grafting of such ovaries orthotopically into mice with intact pituitaries has been shown to result in their development into ovarian tumours in a high proportion of cases within 15 months (Marchant, 1959*b*). But, in the present experiment 2b, grafting to hypophysectomised hosts resulted in their atrophy and degeneration (Fig. 9). Cysts were formed in some cases (Fig. 10) but no tumours occurred, although in the majority of cases there was eventually some evidence of regeneration of pituitary fragments. The process of tumour appearance and growth from preneoplastic ovaries was prevented by the absence of an intact pituitary in the host.

It is concluded that the development of ovarian tumours in mice after DMBA treatment may be divided into two distinct phases. The first phase is one of atrophy resulting from an increase in the rate of atresia of oocytes and follicles in the ovary. It is brought about by the action of DMBA and is quite independent of any action of the pituitary. The second phase of tumour development from such ovaries requires pituitary stimulation of some kind. In most DMBA-treated mice pituitary hyper-stimulation would eventually occur as a result of failure of ovarian hormones from the atrophic ovary.

### SUMMARY

The effect of hypophysectomy on the induction of ovarian tumours in $F_1$ C57Bl × IF mice treated with 7,12-dimethylbenz($\alpha$)anthracene (DMBA) has been studied. The carcinogen proved toxic to hypophysectomised mice. It was possible to show that hypophysectomy did not prevent DMBA treatment from rendering ovaries preneoplastic ; ovaries from such mice were able to develop into tumours when grafted orthotopically into hosts with intact pituitaries. On the other hand, preneoplastic ovaries grafted orthotopically from DMBA-treated to hypophysectomised hosts failed to develop into tumours.

This work was supported by the Birmingham Branch of the British Empire Cancer Campaign.

### REFERENCES

BIELSCHOWSKY, F. AND HALL, W. H.—(1951) *Brit. J. Cancer*, **5**, 331.

BISKIND, G. R. AND BISKIND, M. S.—(1944) *Proc. Soc. exp. Biol. N.Y.*, **55**, 176.—(1948) *Science*, **108**, 137.

CHANG, C. H. AND ECK, G. V. VAN.—(1952) *Cancer Res.*, **12**, 254.

FURTH, J. AND BUTTERWORTH, J. S.—(1936) *Amer. J. Cancer*, **28**, 66.

GARDNER, W. U.—(1950) *Proc. Soc. exp. Biol. N.Y.*, **75**, 434.

HOWELL, J. S., MARCHANT, JUNE AND ORR, J. W.—(1954) *Brit. J. Cancer*, **8**, 635.

JONES, E. C. AND KROHN, P. L.—(1960) *J. Endocrin.*, **20**, 135.—(1961) *Ibid.*, **21**, 497.

KAPLAN, H. S.—(1950) *J. nat. Cancer Inst.*, **11**, 125.

MARCHANT, J.—(1959*a*) *Brit. J. Cancer*, **13**, 652.—(1959*b*) *Acta Un. int. Cancr.*, **15**, 196.—(1960*a*) *Brit. J. Cancer*, **14**, 514.—(1960*b*) *Ibid.*, **14**, 519.

MÜHLBOCK, O.—(1953) *Acta endocr. Copenhagen*, **12**, 47.

SHUBIK, P. AND DELLA PORTA, G.—(1957) *Arch. Path.*, **64**, 691.